# Monitoring changes in body reserves in gestating dairy heifers with 3-dimensional imaging technology: A potential tool to prevent early culling?

**DOI:** 10.3168/jdsc.2025-0830

**Published:** 2025-10-03

**Authors:** Yannick Le Cozler, Laurent Delattre, Thibault Luginbuhl, Maxime Dumesny

**Affiliations:** 1 PEGASE, INRAE, Institut Agro Rennes-Angers, 35590 Saint Gilles, France; 2 3D Ouest, 22300 Lannion, France

## Abstract

The use of 3-dimensional (3D) imaging technology to monitor dairy cows enables the acquisition of morphological, surface, and volume data, which can then be employed to estimate BW or BCS. Accurate tracking of morphological changes in these animals could lead to better management of the gestation and parturition periods, which are especially critical for primiparous cows. Indeed, BCS values that are too low or too high are associated with reproductive disorders and early culling after calving. To assess the potential utility of precise and continuous recordings in gestating animals, we studied BW and morphological changes in 13 gestating Holstein heifers from 3 mo of gestation until the start of lactation. Body condition score was estimated using both 3D imaging technology (BCS3d) and manual scores determined by trained technicians (BCSm). From mid-April (start of experiment) until the end of August (start of calving season) in 2021, average BW increased from 515 (±SD, 40.5) kg to 604 (±49.4) kg, whereas average BCS3d decreased slightly, from 2.50 (±0.19) to 2.32 (±0.30). A similar decrease was noted for BCSm, from 2.63 (±0.19) to 2.11 (±0.24). Linear regression between the 2 estimates of BCS indicated that the technicians usually gave a higher BCS value than the imaging device (BCS3d = 0.97 × BCSm; R^2^ = 0.58). Because of the small number of observations and the fact that the experiment ended after the first calving, we are not able to draw any conclusions about the long-term effects of changes in BCS during gestation on the overall longevity of cows. Nevertheless, using our system, it was possible to detect small changes in BCS during the first gestation of nulliparous cows. Such information could be employed to adapt the diet of heifers to avoid inadequate, insufficient, or excessive fatness at parturition, thus potentially ameliorating calving difficulties (dystocia) and poor starts to lactation. The precise evolution of BCS in the pregnant heifer is of great interest, and the next step will be to examine larger groups of animals to shed more light on the potential value of this technology.

## Highlights

• The use of depth cameras is of increasing interest in managing dairy production.
• Fine changes in the body reserves of gestating heifers are more efficiently detected.
• This detection could be of interest to prevent early culling in primiparous cows.

## Graphical Abstract

**Figure:**
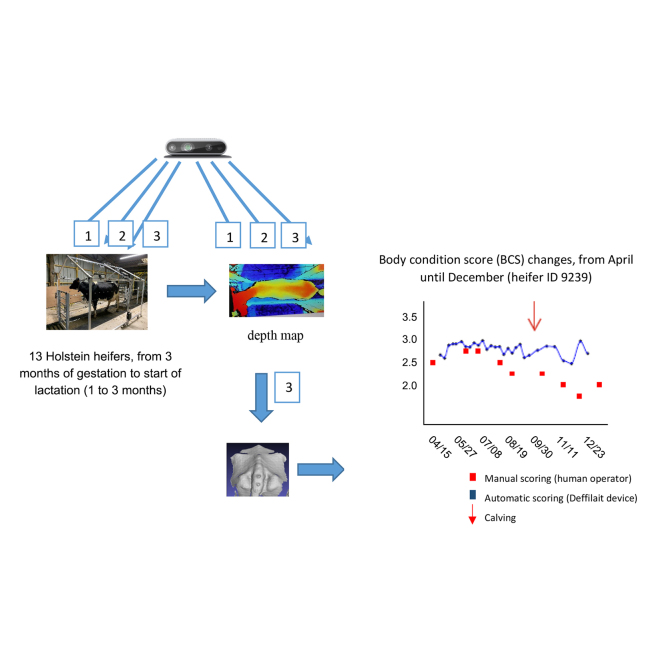
**Summary:** Inadequate body reserves in dairy heifers can be associated with calving problems and, ultimately, premature culling. Body reserves are usually estimated by human evaluators (manual scoring), but frequent measurements are difficult to carry out due to the time required. Fine changes, especially in growing animals, such as heifers, are also hard to detect. The use of depth cameras, with frequent and repeated measurements and relevant analyses, can then be of interest. A comparison between manual (once a month) and automatic scoring (once a week) methods was performed on 13 Holstein heifers (from 3 months of gestation until 1 to 3 months of lactation). Results indicated that the use of depth cameras, with frequent and repeated measurements, makes it possible to more efficiently estimate the evolution of body reserves. Ultimately, this should allow for better monitoring of these animals' diet and body weights to limit the premature culling of dairy heifers.

Body condition score and variants thereof are used on dairy farms to estimate the body reserves of animals and adapt feeding strategies accordingly (Delaby et al., 2010). It is generally estimated by human evaluators based on a visual or manual (palpation) examination. The attributed value is based on references from over 30 years ago (Bazin, 1984; Edmonson et al., 1989), and although there are some differences between references and published grids, it is generally not difficult to convert one form to another (Bédère et al., 2018). Despite the subjectivity of the method, this noninvasive measurement is considered to be the gold standard (Ruegg, 1991) for estimating body reserves of dairy cows and similar indicators. Because it is time-consuming to assess, it is generally recorded no more than once a month on farms, which is typically considered to be enough for the management of dairy cows (Hady et al., 1994). However, in certain cases—such as during pregnancy, particularly the first one—more detailed information on changes in BCS over time could be useful for herd management. To our knowledge, only a limited number of studies have specifically examined the potential benefits of evaluating BCS kinetics in pregnant heifers, which can be different from adult cows in terms of body development and composition. Some studies have used a grid ranging from 1 (very thin) to 9 (obese), with a step of 1 (e.g., Koenen et al., 2001), whereas others have employed a rating from 1 to 5, with a step of 0.5 points (Elanco, 2004). Most often, the grids used for heifers were the same as those for dairy cows, and the results revealed only weak changes in BCS over time, with maximum variations ranging from 0.5 to 1 point. This probably explains why this method is seldom used on growing animals, especially gestating heifers, in which BCS changes can be very hard to detect. Because of this, there is much that we do not understand regarding the possible negative consequences of inadequate feeding management during the first pregnancy for a cow's subsequent health and longevity. From the existing literature, it is clear that BCS values that are too high or low at calving are associated with negative effects on performance (Laven, 2015). Moreover, the number of cows culled in the first lactation is high in France, representing ∼25% of all culled animals, compared with 17% in Ireland, for example (Van Arendonk, 1985; Reproscope, 2019). Reasons for culling are diverse, but problems with reproduction and milk insufficiency are commonly noted (Van Arendonk, 1985). It has been hypothesized that calving difficulties (dystocia) and poor starts to lactation in nulliparous heifers may be the result of inadequate, insufficient, or excessive fattening during gestation, potentially caused or exacerbated by poor feed management (Troccon and Petit, 1989). In this context, data on the precise evolution of BCS during gestation in the pregnant heifer may prove to be of interest for efforts to reduce problems that lead to early culling.

To overcome the difficulties associated with BCS monitoring—namely, that it is time-consuming and subjective—tools based on imaging technologies have been developed for both experimental (Fischer et al., 2015) and commercial purposes (DeLaval, 2025). These technologies are based either on 2-dimensional or 3-dimensional (**3D**) imaging approaches. The methods are nonintrusive; they acquire the external envelope (visible to the naked eye) of a shape and export it in several formats, such as depth maps or point clouds (Le Cozler et al., 2022). To date, numerous studies have used 3D imaging to monitor BCS in dairy cows (Bewley et al., 2008; Fischer et al., 2015; Hansen et al., 2018), but very few, if any, have examined heifers. One reason is because, like weighing equipment, a BCS camera is usually installed on the cow walkway at the entrance or exit of the milking parlor or barn. As a result, these devices are seldom used for heifers. We hypothesized that despite differences in morphology, it would be possible to monitor the evolution of BCS in pregnant dairy heifers using tools initially developed for cows. The main objective of present study was then to confirm that the method presented by Fischer et al. (2015) on cows was validated and easy to use on heifers, using small adjustments of technology (one-shot, automation).

The study was performed from April to December 2021 at the Méjusseaume experimental station of INRAE, Dairy Nutrition and Physiology Unit (**IEPL**; Le Rheu, France; https://doi.org/10.15454/yk9q-pf68), under agreement for animal housing no. C-35-275-23. All animals are reared and managed under routine farm procedures consistent with French animal welfare regulations.

Three-dimensional images of the body shape of animals were acquired and processed using the Deffilait3D device (3D Ouest, Lannion, France), installed at the end of 2020 (Le Cozler et al., 2024). This prototype consists of a fixed metal frame to which 15 RGB-D (Red Green Blue-Depth) sensors are attached (RealSense D415, Intel). The RealSense D415 is a depth camera comprising a pair of depth sensors, a RGB sensor, and an infrared spotlight. This technology is a stereo system that triangulates the depth (distance) for each pixel between the 2 sensors. The infrared projector helps the algorithm to find the correspondence between the 2 images. To reconstruct the image of an animal using a passive stereo system, 2 RealSense D415 cameras, whose relative position and orientation are known, are used to capture the same scene. The 2 images are searched for corresponding pixels, and for each correspondence found, the depth is estimated using camera parameters and triangulation. Finally, all images are combined to reconstruct a 3D image of the entire animal. The full system and its validation are described in Le Cozler et al. (2024). In the present trial, we used only one camera (number 15, the one at the top-back of the device) to assess BCS changes during gestation. Changes in morphological traits (e.g., withers height, chest depth) during the experiment are not discussed here, but the main morphological traits measured at the start of experiment by the device are presented in Table 1.

**Table 1:** Main characteristics of selected heifers at the start of the experiment^1^

Heifer identification	Age (mo)	Gestation stage (mo)	BW (kg)	BCS	Volume (m^3^)	HW (mm)	WH (mm)	CD (mm)	HG (mm)	WB (mm)	Calving date
9209	18.9	2.97	518	2.50	0.582	517	1,421	735	2,012	526	09/23/21
9210	18.9	3.20	518	2.75	0.618	530	1,366	783	2,179	515	09/14/21
9212	18.8	3.14	474	2.5	0.533	470	1,356	725	2,004	479	09/16/21
9213	18.8	2.54	556	3.00	0.617	522	1,359	748	2,185	529	10/11/21
9215	18.8	2.74	554	2.75	0.611	552	1,431	806	2,111	520	10/02/21
9221	18.63	2.74	532	2.50	0.622	523	1,413	763	2,222	473	10/09/21
9224	18.6	3.07	475	2.25	0.534	507	1,448	761	1,992	509	10/16/21
9230	18.5	2.97	504	2.25	0.567	482	1,410	783	2,107	489	09/29/21
9238	18.4	3.14	564	3.00	0.653	516	1,396	794	2,260	532	09/18/21
9239	18.4	2.84	490	2.50	0.556	496	1,297	738	2,058	521	10/02/21
09244	18.2	3.00	516	2.75	0.613	509	1,326	750	2,142	490	10/27/21
9252	18.0	3.10	418	2.25	0.514	508	1,340	701	2,020	521	09/24/21
9270	17.3	2.61	448	2.50	0.536	461	1,324	731	1,954	481	10/10/21
Average	18.5	2.93	505	2.58	0.581	507	1,376	755	2,096	506	—
SD	0.46	0.21	43	0.26	0.581	25	47	30	97	21	—

1 BCS according to Bazin (1984); HW = hip width; WH = withers height; CD = chest depth; HG = heart girth; WB = buttocks width.

Because this study was designed to investigate the potential utility of this methodology for this particular application, we focused on a limited number of animals, sufficient for revealing the strengths and weaknesses of this approach while avoiding an unnecessarily large workload and the collection of a large amount of data that might not be useful in the future. A total of 13 pregnant Holstein heifers, chosen to be heterogeneous in terms of BW, morphological development, and body reserve content (estimated through BCSm; see below), were selected on average one month before the start of the experiment (April 15, 2021) and followed until calving (Table 1). The experiment started at least 3 mo after the start of gestation to avoid possible abortion due to excessive handling and stress caused by the experiment.

Heifers were turned out to pasture in March and received no feed except for grass, along with supplemental vitamins and minerals. Three weeks before the expected date of calving, heifers were group-housed with multiparous cows and fed accordingly.

Each week, five 3D images were recorded per heifer using the Deffilait3D device, with the goal of obtaining at least one of very good quality (score 3+ or 4+, with + meaning the entire animal with the head; see Le Cozler et al., 2024 for more information). In the end, from the 5 images recorded per heifer, at least 3 good-quality images were obtained. The images from camera 15 were then used to estimate the average BCS (**BCS3d**) according to the method developed by Fischer et al. (2015). This method can be assumed as follows: (1) normalization of the 3D surface from 4 anatomical landmarks (top of the left and right ischia), basis of the left and right sacra), extraction of a region of interest (hips–tips region) sampled on a 150 × 150 pixels grid; (2) shape analysis by principal component analysis (**PCA**) and then then multiple linear regression of the PCA coordinates to predict the BCS. This method has been validated on cows with a root mean square error of prediction of 0.31 to 0.32 BCS points and substantially better repeatability and reproducibility than manual BCS. The hypothesis was that the statistical model obtained on cows could be generalized to heifers without recalibrating the database, even if input data type changed, using a single depth image as input instead of a reconstruction using the Kinect fusion algorithm. The detection of the 4 points was also automatized so that the measurement could be obtained directly from the depth images without manual pointing.

Meanwhile, manual measurements of BCS (**BCSm**) were carried out once a month (until the end of December) according to the methodology developed by Bazin (1984). In this experimental facility, specially trained and dedicated technicians routinely estimate BCS (BCSm) once a month on all cows. Because manual measurement is still considered to be the reference method (the gold standard), we used these data to compare the BCS values from the 2 methods. At the same time (i.e., every month) BW was recorded using a weighing scale, but during lactation, measurements were taken 3 times a week on average. Weighing usually occurred between 1000 and 1200 h to maintain consistency in the conditions. The number of BW available varied then from 26 to 49 and in total, 29 BCS3d (once a week) and 8 to 10 BCSm were recorded per animal. Indeed, some BCSm and BW values were considered to be abnormal (BCS difference of more than 0.5 points, or 20% changes in BW from previous and subsequent animal's value) and were not kept.

As explained previously, for this pilot study, we deliberately restricted the number of observations, and only descriptive analyses were performed. Data were visualized using the ggplot2 package (v. 3.2.1; Wickham et al., 2019) of R software (R Core Team, 2019). In addition to studying BCS changes, we also tested a data-smoothing method for taking into account missing or aberrant data, which are common in commercial herds. Using this method, it was possible to assign a value close to reality. For this, we used LOESS (locally estimated scatterplot smoothing) regression, a nonparametric regression method (i.e., it is not associated with an equation, such as a classic linear or polynomial regression) that makes it possible to produce smoothed curves fitted to a point cloud (blue curves on Figure 1; Cleveland, 1979).

**Figure 1:**
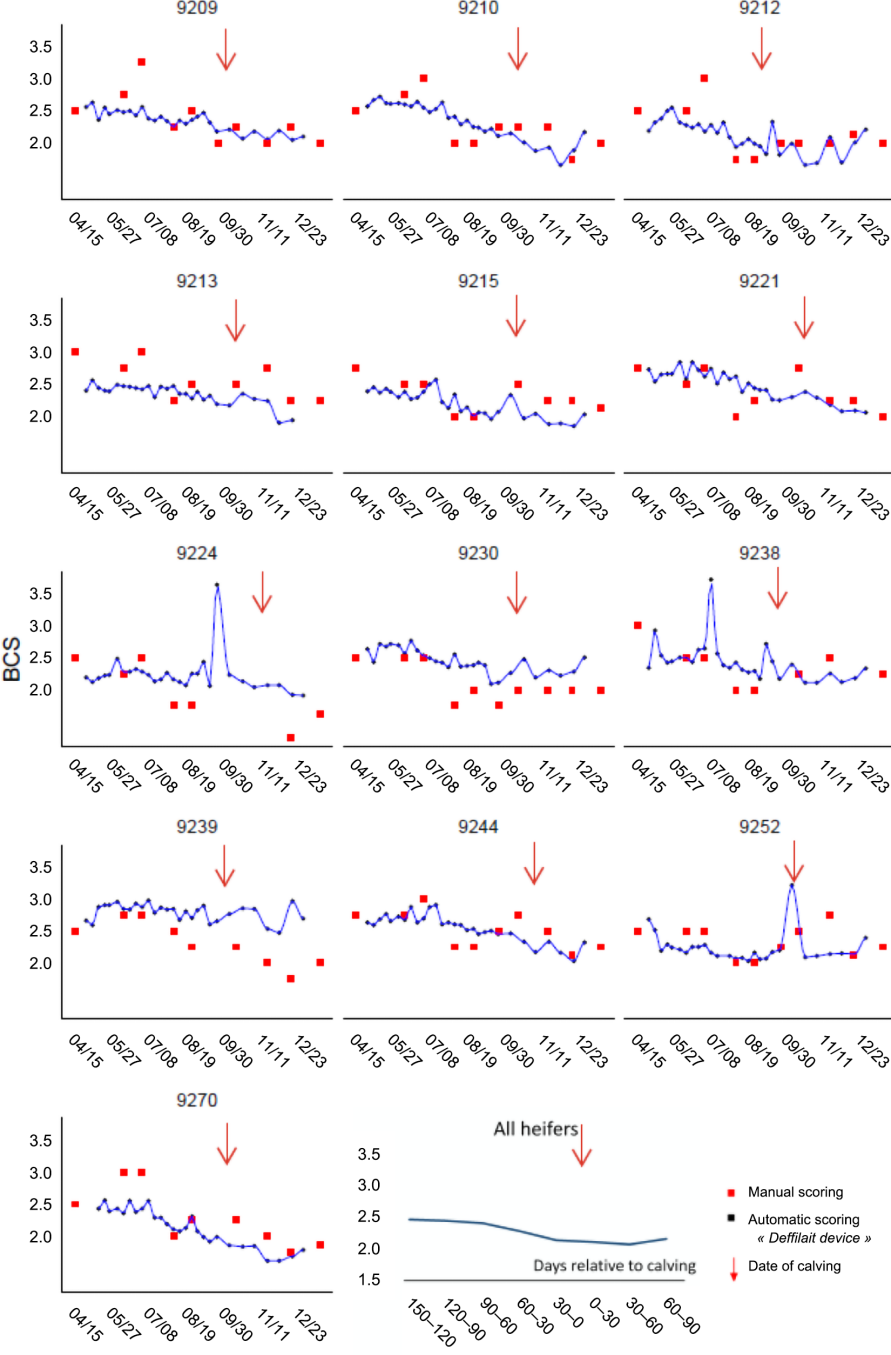
Changes in BCS over time in 13 gestating heifers.

During the experiment, changes in BCS (both BCSm and BCS3d) of the 13 heifers were limited, as expected. Comparison of BCS3d (black dots in Figure 1) and BCSm (red dots in Figure 1) showed that the 2 sets of values were very close.

From April (start of experiment) until the end of August (start of calving season), average BCS3d (±SD) decreased slightly, from 2.50 (±0.19) to 2.32 (±0.30), whereas BW increased from 515 (±40.5) kg to 604 (±49.4) kg (not presented). After calving and until the end of experiment (December 31), BCS still decreased in most cows. On the contrary, most of them gained BW.

The decrease in BCS value was also noted in BCSm estimations (from 2.63 ± 0.19 to 2.11 ± 0.24). The higher frequency of BCS estimations from the Deffilait3D device (weekly vs. monthly) made it possible to highlight slight variations in the fattening state of heifers that would be invisible to the naked eye. Linear regression between the 2 estimates of BCS indicated that the technicians usually gave a higher BCS value than the one calculated from the device (BCS3d = 0.97 × BCSm; R^2^ = 0.58). However, the correlation is not very high, with important variations.

Our analyses revealed that, even as the BW of the 13 Holstein heifers increased during the course of gestation, their fattening state (BCS) decreased slightly. The gain in BW during gestation was in the range of what was expected and has been described in the literature. The BCS values at calving appeared slightly lower than those expected or recommended for primiparous Holstein cows, but in line with what was expected from this experimental herd. Moreover, although absolute BCS scores are useful indicators for breeding, for many stakeholders, it is the evolution of the fattening state that is typically more relevant. This kind of information has already demonstrated its usefulness in adult cows (Delaby et al., 2009), but these data are not commonly recorded in heifers, especially during gestation. Indeed, although previous research has examined the evolution of BW in heifers (Troccon and Petit, 1989), information on the fine-scale evolution of BCS values, assessed manually or via a 3D imaging device, is still not widely available. With our approach, in which BCS was estimated once a week using a 3D imaging device, we were able to detect fine-scale variations (increments of 0.1 to 0.15 points) that were not visible in the monthly scores assigned by the technicians (increments of 0.25 or 0.5 points).

Changes in BCS in gestating heifers are generally low, with a maximum gain of 0.5 to 1 point, and sometimes no change at all. Here, the gains we observed were close to zero or even negative. In a previous experiment (Le Cozler et al., 2019a), we noted BCS gains of 0.1 and 1 point in 2 groups of late first-calving animals (36 mo of age on average) in Holstein heifers that respectively reached either 479 g/d or 892 g/d of growth from 4 mo of gestation until 3 wk before calving. In the present study, ADG was 665 g/d, without any visible effect on BCS gain. The likely explanation for this is that young gestating heifers (calving at 24 mo) still require substantial resources for growth, both their own and of the calf in utero; if their feed allowance is not adequate or sufficient, animals may need to mobilize their own body reserves to compensate. With detailed, real-time information on changes in body reserves, it becomes possible to adapt the feeding recommendations for pregnant heifers to limit the risks at calving, ensure the optimal development of the fetus, and prepare the mother for a good first lactation. Previous studies were mainly performed on lactating cows (Hallén Sandgren and Emanuelson, 2016), but the relationships between BCS changes during the last two-thirds of gestation in heifers and subsequent performances, in primiparous cows especially, are not well documented. Combined with the measurements of other changes such as weight or morphological traits, the Deffilait3D device is then opening interesting avenues for further investigations.

The results of the present study indicate that the Deffilait3D device can be used to estimate BCS in young animals, in addition to generating data on morphology and estimating BW (Le Cozler et al., 2019b). With a comparable Deffilait3D system on-farm, Lebreton et al. (2023) confirmed that it is possible to obtain information on morphological traits quickly, frequently, and from a large number of growing beef animals. Xiong et al. (2023) also demonstrated the interest of depth images to estimate BW and BCS on mature beef cows and Xavier et al. (2024) used 3D images to estimate the body composition of growing bulls. All of these results highlight the promise of 3D imaging technologies for the future of dairy and beef production. In addition, it is likely that these technologies can be successfully applied in other production systems, such as with dairy goats and ewes.

This study showed that it was possible to closely monitor changes in the fattening status of heifers. This will make it possible to track any links between this status and calving difficulties or unsatisfactory early lactation. This may result in early culling of animals, and although it was obviously beyond the scope of this study to establish a link between changes in BCS during this first gestation and the longevity of the animals, it nevertheless opens up new perspectives. We had only a limited number of observations and ended the experiment after first calving, and, moreover, none of the 13 heifers had any calving problems. Our main aim was to demonstrate that it was possible to detect such changes during the first gestation of nulliparous cows. We did not modify any aspect of animal management during this period; all animals were fed and housed together. The fact that changes in BCS were detected, even small ones, suggests that in the future, it may be feasible to use this type of monitoring to adapt cows' feeding regimens during gestation, thus avoiding inadequate, insufficient, or excessive weight gain. It has long been known that inadequate fattening can lead to calving difficulties (dystocia) and poor starts to lactation (Troccon and Petit, 1989). A tool that can help illuminate the precise evolution of BCS during gestation in the pregnant heifer may thus help to preserve animal health and prevent early culling.

This study demonstrates that it is possible to use 3D imaging technology to detect and track fine-scale variations in BCS in pregnant heifers. Changes in BCS are considered to be more important for management of animals than absolute value of BCS itself, as it can be operator dependent. Such changes are generally invisible with the traditional manual method, and are not easy to manage given the rapid weight gain that occurs in this period (up to 100 kg of BW in the second and third trimester of gestation). The present study was carried out on a limited number of heifers, and it will be necessary to conduct additional research with a larger number of heifers and over a longer period in order to support these preliminary results. Such studies will open opportunities for studying the effect of early-life management not only on early culling, but on the longevity of dairy cows more generally.
